# Concurrent Control of Sodium and Bicarbonate Serum Concentrations Using a Four‐Stream Hemodialysis Fluid Delivery System

**DOI:** 10.1111/hdi.13205

**Published:** 2025-03-06

**Authors:** Deepak Malhotra, Susie Q. Lew, Raymond E. Garrett, Ramin Sam, Robert H. Glew, Todd S. Ing, Antonios H. Tzamaloukas

**Affiliations:** ^1^ Department of Medicine University of Toledo School of Medicine Toledo Ohio USA; ^2^ Department of Medicine George Washington University Washington District of Columbia USA; ^3^ Trauma Research, Swedish Medical Center Englewood Colorado USA; ^4^ Department of Medicine Zuckerberg San Francisco General Hospital and the University of California in San Francisco School of Medicine San Francisco California USA; ^5^ Department of Surgery University of New Mexico School of Medicine Albuquerque New Mexico USA; ^6^ Department of Medicine Stritch School of Medicine, Loyola University Chicago Maywood Illinois USA; ^7^ Research Service, Department of Medicine Raymond G. Murphy Veterans Affairs Medical Center, University of New Mexico School of Medicine Albuquerque New Mexico USA

**Keywords:** four‐stream dialysis system, hemodialysis, hypernatremia, hyponatremia, metabolic acidosis, metabolic alkalosis

## Abstract

**Background:**

Previously, two reports proposed a four‐stream dialysis fluid delivery system consisting of an acid concentrate, a base concentrate, a sodium chloride concentrate, and product water for correcting dysnatremias and metabolic acid–base disorders separately, by hemodialysis.

**Methods:**

This report describes a new method for the clinical use of the previously reported four‐stream dialysis fluid delivery system to treat concurrently dysnatremias and metabolic acid–base disturbances by hemodialysis. Pumps attached to each concentrate are designed to control its flow rate. Formulas were derived to determine the flow rate of each of the pumps controlling the flows of the product water (***W***), the base concentrate (***B***), and the sodium chloride concentrate (***S***) for any prescribed combination of sodium and bicarbonate concentrations is the final dialysis fluid. In this scheme, the flow rate of the acid concentrate (***A***), the concentrations of its contents in the final dialysis fluid remain constant. The flow rate ratio ***W*:*S*:*B*:*A*** remains also constant at 45 (i.e., 45X).

**Results:**

The formulas were entered in an EXCEL flow sheet which determines the flow rate ratio ***W*:*S*:*B*:*A*** for any desired combination of sodium and bicarbonate concentrations in the dialysis fluid. The upper and lower limits of the concentrations of sodium and bicarbonate in the dialysis fluid were computed. The system has not been applied clinically. Measurements of any electrolyte concentrations have not been made.

**Discussion:**

This system makes the treatment of profound dysnatremias, metabolic acid–base disorders, and combined dysnatremias and metabolic acid–base disorders feasible. The clinical application of the system demands prior in vitro or ex vivo studies plus fastidious and expert attention to ensure safe and dependable application.

## 1 Introduction

Dysnatremias and acid–base disturbances are common and have adverse outcomes in patients undergoing hemodialysis for end‐stage kidney disease [1, 2, 3, 4] or for severe acute kidney injury [5, 6]. Correction of dysnatremias or acid–base disturbances by hemodialysis requires the appropriate composition of the dialysis fluid [7, 8]. Mathematical models are used to determine the required change in the dialysis fluid composition in this case [9]. Adjustments of the dialysis fluid concentrations of either sodium or bicarbonate in systems that employ separate streams of product water (***W***), bicarbonate concentrate (***B***), and acid concentrate (***A***) for constitution of the dialysis fluid alter the concentrations of the ingredients of the acid concentrate in the dialysis fluid, for example, potassium and calcium [10].

We had earlier proposed a dialysis fluid delivery system consisting of four different streams, specifically product water (***W***), sodium chloride concentrate (***S***), sodium bicarbonate concentrate (***B***), and acid concentrate (***A***), with a sum of the ***W*:*S*:*B*:*A*** flow rates equal to 45 (designated 45X) [11, 12]. In this novel four‐stream system, the “normal” flow rate ratio ***W*:*S*:*B*:*A*** of 40.56:1.72:1.72:1, totaling 45 (45X), provides dialysis fluid and bicarbonate concentrations equal to 137 and 37 mmol/L respectively [11, 12].

How to calculate the composition of the dialysis fluid for any desired value of sodium and bicarbonate dialysis fluid concentrations, combinations of these two concentrations, and the limits of these two concentrations were not provided in the reported presentations of the novel system [11, 12]. Instead, in the reported presentations of the novel system, dialysis fluid sodium concentration is modified when a change in the flow rate of stream ***S*** is offset by an equal change of stream ***W*** in the opposite direction. An electronic pulley‐like mechanism situated between the ***S*** and ***W*** streams was proposed for this modification of dialysis fluid sodium [11]. This application of the four stream system calculates the change in dialysis fluid sodium after specified changes in the flows of streams ***S*** and ***W*** but does not calculate the flow rate ratio ***W*:*S*:*B*:*A*** for a required value of sodium [11]. Similarly, modification of dialysis fluid bicarbonate concentration is achieved when a change in the flow rate of stream ***B*** is offset by an equal change in the opposite direction of the flow rate of stream ***S*** by an electronic pulley‐like mechanism situated between streams ***B*** and ***S*** and this application does not address how to obtain a specific value of dialysis fluid bicarbonate [12]. The total flow rate of 45X does not change with the changes in the flow rates of ***W***, ***S***, and ***B*** described above. Furthermore, since the flow rate of stream ***A*** remains constant, the concentrations of the components of this stream in the final dialysis fluid also do not change [11, 12].

The aim of the present report is to present a new method that addresses all three of the requirements for the clinical application of the proposed dialysis system. Specifically, this new method calculates the changes in the flow rate ratio ***W*:*B*:*S*:*A*** that is required for obtaining desired values of both sodium and bicarbonate concentrations in the dialysis fluid by this novel four‐stream system. In this new method, a machine with the appropriate computer software can control the flow rate of each stream ***W***, ***S***, ***B***, and ***A***. The flow rates of the streams required for specific concentrations of sodium and bicarbonate in the dialysis fluid are calculated by formulas. The precision of the pumps, which is critical for success of the method, should be tested by measurement of sodium and bicarbonate concentrations in the dialysis fluid after each change in the dialysis prescription. This method is designed to achieve the desired level of correction of dysnatremias or metabolic acid–base disorders, plus simultaneous correction of dysnatremias and acid–base disturbances. In addition, this method computes the determination of the ranges of the concentrations of sodium and bicarbonate allowed by this four‐stream system.

## 2 Methods

### 2.1 Development of Formulas Determining the Flow Rate Ratio *W*:*S*:*B*:*A*

**FIGURE 1:**
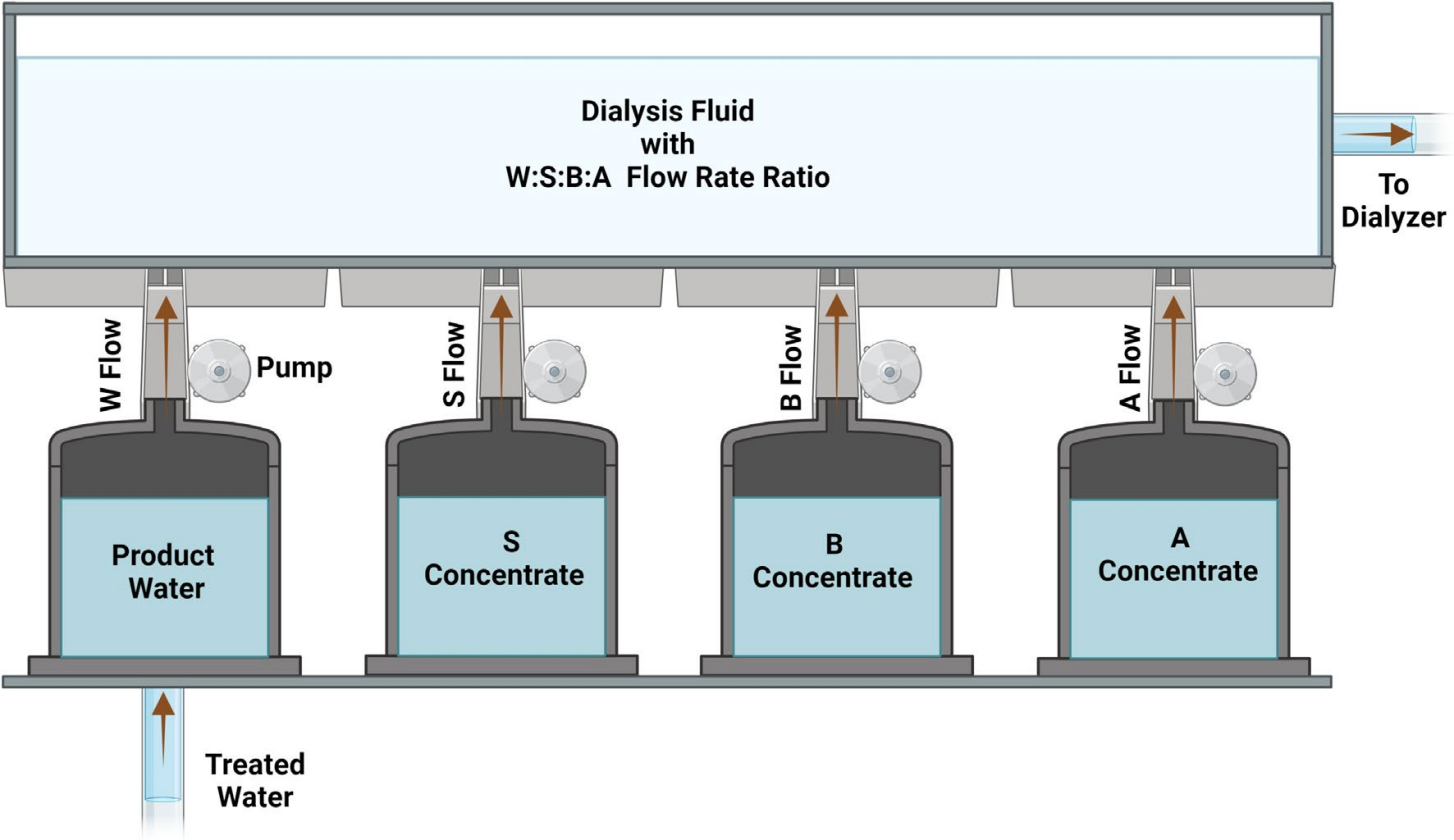
Concept of composition of ***W***: ***S***: ***B***: ***A*** hemodialysis fluid by flow rates of its four components. Each stream has its own separate pump. The flows through the pumps of streams ***B***, ***S***, and ***W*** are calculated by the text formulas 2, 4, and 5, respectively, for any combination of desired values of bicarbonate and sodium concentrations in the dialysis fluid. The flow rate of stream ***A*** is always 1 and the flow rate ratio ***W***: ***S***: ***B***: ***A*** is always 45 (formula 5). The in vitro and ex vivo studies prior to the clinical application of the system should address among other items the types of pump types and tanks as well as the relative speeds of the pumps. Created with BioRender.com. ***A*** = acid stream; ***B*** = sodium bicarbonate stream; ***S*** = sodium chloride stream; ***W*** = product water stream.

Table 1 specifies the concentrations of the ingredients in the concentrates ***S***, ***B*** and ***A***. Figure 1 shows a scheme of the proposed system. The flow rates of stream ***B*** for any desired concentration of bicarbonate and of stream ***S*** for any desired concentration of sodium in the final dialysis fluid at the ratio ***W*:*S*:*B*:*A*** of 45 (45X) are calculated using the formulas presented subsequently. The flow rate of stream ***A*** remains constant at 1.0. Finally, the flow rate of stream ***W*** is adjusted so that the ratio ***W*:*S*:*B*:*A*** remains constant at 45 (45X).

**TABLE 1:** Concentrations, in mmol/L, of the ingredients of the sodium chloride concentrate (***S***), the sodium bicarbonate concentrate (***B***), and the acid concentrate (***A***) of the proposed 45X, four‐stream, bicarbonate‐based dialysis fluid.

Ingredients	Sodium chloride concentrate (*S*)	Bicarbonate concentrate (*B*)	Acid concentrate (*A*)
Sodium chloride (NaCl)	968	—	2835
Sodium bicarbonate (NaHCO_3_)	—	968	—
Potassium chloride (KCl)	—	—	90
Calcium chloride (CaCl_2_.2H_2_O)	—	—	56.25^a^
Magnesium chloride (MgCl_2_.6H_2_O)	—	—	16.875
Dextrose (C_6_H_12_O_6_.H_2_O)	—	—	252
Acids			
Acetic acid (CH_3_COOH) Or	—	—	180
Citric acid (C_6_H_8_O_7_) Or	—	—	108
Sodium diacetate (NaH{C_2_H_3_O_2_}_2_)	—	—	180

*Note*: Reproduced with permission from Lew et al. [12].

^a^ The concentration of CaCl_2_ may be modified according to patient needs. Changing this concentration will change also the chloride concentration in the dialysis fluid.

In addition to supplying several of the dialysis fluid ingredients, stream ***A*** allows adjustment of the pH of the dialysis fluid, which would be dangerously high if only streams ***W***, *S*, and ***B*** were operating [13]. Dialysis fluid pH values higher than 7.8 cause precipitation of ingredients of the dialysis fluid (e.g., calcium) [4, 9, 13]. Low levels of dialysis fluid pH have been associated with blood clotting in the dialyzer [14]. To optimize safety, the pH of the dialysis fluid should ideally be between 6.8 and 7.2 [12]. Chemical reaction between the sodium bicarbonate in stream ***B*** and any of the three acids in stream ***A*** lowers the final concentration of bicarbonate and adds dissolved carbon dioxide—and carbonic acid—to the dialysis fluid, thereby maintaining the pH within the safe range [7, 12]. After transfer into the blood and metabolism of the acid anion of stream ***A*** to carbon dioxide and water, the sodium ion gained as sodium acetate or sodium citrate reconstitutes the buffer base lost in the dialysis fluid with the titration in the dialysis fluid of sodium bicarbonate by the acid component in stream ***A*** [10].

Table 2 shows the concentrations of electrolytes delivered by streams ***S***, *B*, and ***A*** to the final dialysis fluid, plus the concentrations of dextrose and acid delivered to this fluid by stream ***A*** when the ratio ***W*:*S*:*B*:*A*** is 42:1:1:1. In this case, the concentrations of sodium and chloride delivered to the final dialysis fluid through stream ***S*** plus the concentrations of sodium and bicarbonate delivered through stream ***B*** are all 21.5 mmol/L. These concentrations of 21.5 mmol/L are used to calculate the required flow rates of streams ***W***, ***S***, and ***B*** for any desired concentrations of sodium and bicarbonate and for the corresponding concentration of chloride in the dialysis fluid.

**TABLE 2:** Concentrations, in mmol/L, of ions, dextrose and acids, all in mmol/L, delivered to the final 45X dialysis fluid by the sodium chloride stream (***S***), the sodium bicarbonate stream (***B***), and the acid stream (***A***) at a ***W***: ***S***: ***B***: ***A*** flow rate ratio of 42:1:1:1.

Ingredients	Sodium chloride stream (*S*)	Sodium bicarbonate stream (*B*)	Acid stream (*A*)
Sodium	21.5	21.5	63^a^
Bicarbonate	—	21.5	—
Chloride	21.5	—	68.25
Potassium	—	—	2
Calcium	—	—	1.25
Magnesium	—	—	0.375
Dextrose	—	—	5.6
Acids			
Acetic acid Or	—	—	4
Citric acid Or	—	—	2.4
Sodium diacetate	—	—	4

^a^ Sodium concentration provided by a stream ***A*** flow rate of 1 to the final 45X dialysis fluid is 67 mmol/L when sodium diacetate is used instead of acetic acid or citric acid. Since this system is a 45X one, if one divides a given number in Table 1 by 45, one will arrive at the corresponding dialysis fluid level depicted in Table 2, with the exception of the chloride concentration delivered by stream ***A*** (note that 1 mmol/L of calcium chloride or magnesium chloride contains 2 mmol/L of chloride).

The concentration of sodium delivered to the final 45X dialysis fluid by stream ***A*** does not change with different ***W***: ***S***: ***B***: ***A*** ratios because the flow rate of stream ***A*** is constant. Calculation of the flow rate ratio ***W***: ***S***: ***B***: ***A*** for any desired value of [*Na*]_df_ requires accounting for the dialysis fluid concentration of sodium provided by stream ***A***. The flow rates for streams ***B***, ***S***, and ***W*** for any desired bicarbonate and sodium dialysis fluid concentrations are calculated using the formulas contained in Table 3. Bicarbonate is contained in stream ***B***. The desired bicarbonate concentration is the product of the concentration of bicarbonate delivered to a dialysis fluid volume of 45 L by a flow rate ***B*** equal to 1 (21.5 mmol/L) times the unknown flow rate ***B*** (formula 1). The desired flow rate ***B*** for any specific value of dialysis fluid bicarbonate concentration is obtained by solving formula 1 for ***B*** (formula 2).

**TABLE 3:** Formulas for calculating the desired [*Na*]_df_ and [*HCO*_3_]_df_.

${\left[\mathrm{HC}O_{3}\right]}_{\mathrm{df}}=B\times 21.5$ (1)
$B=\text{Desired}\;{\left[\mathrm{HC}O_{3}\right]}_{\mathrm{df}}/21.5$ (2)
${\left[\mathrm{Na}\right]}_{\mathrm{df}}=S\times 21.5+\text{Desired}\;{\left[\mathrm{HC}O_{3}\right]}_{\mathrm{df}}+63\;\left(3\right)$
$S=\left(\text{Desired}\;{\left[\mathrm{Na}\right]}_{\mathrm{df}}-\text{Desired}\;{\left[\mathrm{HC}O_{3}\right]}_{\mathrm{df}}-63\right)/21.5$ (4)
$W=45-\left(S+B+1\right)$ (5)
${\left[\mathrm{Cl}\right]}_{\mathrm{df}}=S\times 21.5+68.3$ (6)

Abbreviations: [*Cl*]_df_ = chloride concentration in the dialysis fluid; [*HCO*_*3*_]_df_ = bicarbonate concentration in the dialysis fluid; [*Na*]_df_ = sodium concentration in the dialysis fluid.

Sodium is present in streams ***S***, ***B***, and ***A***. Flow rates of all three streams must be included in the calculation of the flow rate ratio ***W***: ***S***: ***B***: ***A*** for any desired dialysis fluid sodium concentration. The sodium concentration delivered to the dialysis fluid by stream ***A*** is 63 mmol/L (Table 2). Any flow rate of stream ***B*** will contain equal concentrations of bicarbonate and sodium (Tables 1 and 2). Therefore, the numerical value of the desired bicarbonate concentration can be used in the calculation of the flow rate of stream ***S*** as the concentration of sodium provided by stream ***B*** to the final dialysis fluid. Formula 3 calculates the desired dialysis fluid sodium concentration. The desired flow rate ***S*** for any specific concentration of sodium in the dialysis fluid is obtained by solving formula 3 for ***S*** (formula 4). When stream ***A*** contains sodium diacetate instead of acetic acid or citric acid, 67 should be substituted for “63” in formula 4.

The flow rate of stream ***W*** required for keeping the total ***W***: ***S***: ***B***: ***A*** flow rate at 45 is calculated by formula 5. Chloride is present in streams ***S*** and ***A***. The concentration of chloride provided to the final dialysis fluid by stream ***A*** is 68.3 mmol/L (Table 2). The concentration of chloride in the dialysis fluid is calculated by formula 6.

## 3 Results

### 3.1 Calculations of *W*:*S*:*B*:*A* Flow Rates by This Method

An EXCEL spread sheet allowing calculation of the ***W***: ***S***: ***B***: ***A*** flow rates and of dialysis fluid chloride for any desired combination of sodium and bicarbonate concentrations in the dialysis fluid by formulas 2, 4,5, and 6 is provided. The results presented in this section contain only calculations in hypothetical patients. Application of the proposed method in actual patients and measurements of electrolyte concentrations have not been performed. Table 4 shows representative ***W***: ***S***: ***B***: ***A*** ratios for a wide range of combinations of desired dialysis fluid sodium and bicarbonate concentrations and the corresponding dialysis fluid chloride values.

**TABLE 4:** Flow rates of product water (***W***), sodium chloride stream (***S***), bicarbonate stream (***B***), and acid stream (***A***), for various combinations of sodium and bicarbonate concentrations plus the corresponding chloride concentrations in the final 45X dialysis fluid.

[*Na*]_df_ ^a^	107	127	137	147	157	107	117	137	147	157
[*HCO*_*3*_]_df_ ^b^	37	37	37	37	37	17	17	17	17	17
[*Cl*]_df_	75.3	95.3	105.3	115.3	125.3	95.3	105.3	125.3	135.3	145.3
*W* flow	41.95	41.02	40.56	40.09	39.63	41.95	41.49	40.56	40.09	39.63
*S* flow	0.33	1.26	1.72	2.19	2.65	1.26	1.72	2.65	3.12	3.58
*B* flow	1.72	1.72	1.72	1.72	1.72	0.79	0.79	0.79	0.79	0.79
*A* flow	1.00	1.00	1.00	1.00	1.00	1.00	1.00	1.00	1.00	1.00
[*Na*]_df_ ^a^	107	127	137	147	157	107^c^	117	137	147	157
[*HCO*_*3*_]_df_ ^b^	27	27	27	27	27	47^c^	47	47	47	47
[*Cl*]_df_	85.3	105.3	115.3	125.3	135.3	—	75.3	95.3	195.3	115.3
*W* flow	41.95	41.02	40.55	40.09	39.63	—	41.49	40.56	40.09	39.62
*S* flow	0.79	1.72	2.19	2.65	3.11	—	0.32	1.25	1.72	2.19
*B* flow	1.26	1.26	1.26	1.26	1.26	—	2.19	2.19	2.19	2.19
*A* flow	1.00	1.00	1.00	1.00	1.00	—	1.00	1.00	1.00	1.00

*Note*: [*Na*]_df_ (sodium concentration in the dialysis fluid), [*HCO*_3_]_df_ (bicarbonate concentration in the dialysis fluid) and [*Cl*]_df_ (chloride concentration in the dialysis fluid) are in mmol/L. Column in bold text represents the “normal” composition and flow rate ratios of the dialysis fluid [10, 11]. The concentrations of the ingredients of flow rate ***A*** in the dialysis fluid are those shown in Table 2 because flow rate ***A*** is 1.00 in all flow rate ratios.

^a^ At the same ***W***: ***S***: ***B***: ***A*** flow rate ratios, dialysis fluid sodium values will be 4 mmol/L higher than the values in each column when sodium diacetate is used in stream ***A***.

^b^ Value of bicarbonate in the dialysis fluid prior to any chemical reactions. Actual value of dialysis fluid bicarbonate will be lower after titration of the sodium bicarbonate by the component of stream ***A***.

^c^ The lowest dialysis fluid sodium that is allowed by a dialysis fluid bicarbonate equal to 47 is 110 mmol/L.

### 3.2 Ranges of Dialysis Fluid Sodium and Bicarbonate Concentrations Allowed

The ranges of sodium and bicarbonate concentrations in the dialysis fluid that can be obtained with any hemodialysis system dictate the ranges of dysnatremias and metabolic acid–base disorders that can be treated by this system. Commercially available four‐stream hemodialysis systems can obtain a 130–160 mmol/L sodium concentration range [11, 15]. The upper limit of the sodium range in the four‐stream system presented in this report is considerably higher than any clinical requirement might demand. The lower limit of this range represents the major advantage offered by this new system. With this system the sodium concentration reaches its lower limit when the flow rate of stream ***S*** becomes zero. From formula 4, the flow rate of stream ***S*** becomes zero when sodium concentration is equal to bicarbonate concentration plus 63. Thus, the lower limit of sodium concentration is determined by the desired bicarbonate concentration. For example, the lower limit of sodium concentration is 100 mmol/L when bicarbonate is 37 and 90 mmol/L when bicarbonate concentration is 27 mmol/L.

The upper limit of bicarbonate concentration is reached when the flow rate of stream ***S*** becomes zero and is therefore dependent on the desired concentration of sodium. For example, the upper limit of the range of bicarbonate concentration is 74 mmol/L at a sodium concentration of 137 mmol/L, 37 mmol/L at a sodium of 100 mmol/L, and 27 mmol/L at a sodium of 90 mmol/L. The lower value of bicarbonate concentration that can be obtained by this system is zero when the flow rate of stream ***B*** is zero.

### 3.3 Safe Range of Dialysis Fluid Bicarbonate Concentration

The safe range of the bicarbonate concentrations corresponding to a dialysis fluid pH range of 6.8–7.2 is calculated as follows: When stream ***A*** contains acetic acid with a concentration of 4 mmol/L in the dialysis fluid (Table 2), titration of this acetic acid by bicarbonate will result in a sum of concentrations of carbonic acid and dissolved carbon dioxide very close to 4 mmol/L. [7, 16] Conversion of 4 mmol/L of carbonic acid plus dissolved carbon dioxide, which represents by far the greatest part of the 4 mmol/L, to partial pressure of carbon dioxide (*P*_*CO*2_) using a conversion coefficient of 0.0301 mmHg per mmol/L [17] results in a *P*_*CO*2_ of 4/0.0301 = 133 mmHg. For a temperature of 37°C, the dissociation constant (pK) of carbonic acid is 6.108 at a pH of 7.2 [17], and 6.199 at a pH of 6.8 [18]. The bicarbonate values that correspond to pH values of 7.2 and 6.8 at a *P*_*CO*2_ of 133 mmol/L, calculated by the formula 0.0301 × *P*_*CO*2_ × 10^(pH–pK)^ which is derived by solving the Henderson–Hasselbalch formula for bicarbonate [17], are 4 × 10^(7.2–6.108)^ = 49.4 mmol/L for a pH of 7.2 and 4 × 10^(6.8–6.199)^ = 16.0 mmol/L for a pH of 6.8. One point of caution for choosing the low value of the range of dialysis fluid bicarbonate concentration consists of the fact that the carbonic acid pK of 6.199 for a pH of 6.8 was obtained from a linear regression of the pK on pH at pH values between 7.0 and 7.6 on pK at 37°C [18], not from experimental data. Measuring the pH of the dialysis fluid after each change in its composition is required.

### 3.4 Prescription of the Sodium and Bicarbonate Concentrations in the Dialysis Fluid

The prescription of sodium concentration in the dialysis fluid for correction of any dysnatremia requires the following steps: (a) Pseudohyponatremia should be ruled out [19]. (b) Osmotic demyelination [20, 21] should be prevented by choosing the target dialysis fluid sodium concentration. The target rise in serum sodium concentration for treating hyponatremia with infusion of hypertonic saline was set at 4–6 mmol/L. [22] Larger increase in serum sodium, up to 12 mmol/L, in a hemodialysis session, appears to be safe [2, 23]. (c) The effects of the Gibbs–Donnan equilibrium principle on the sodium concentrations in serum, which contains polyanions, and in dialysis fluid, which does not, should be accounted for in the prescription of the dialysis fluid sodium concentration [24, 25]. (d) Changes in the external balances of sodium, water, and potassium which affect the change in serum sodium concentration during treatment of hyponatremia, other than the changes from a hemodialysis session, should be accounted for [26].

The prescription of the dialysis fluid bicarbonate concentration requires accurate diagnosis of the underlying acid–base disorder(s). Although metabolic acidosis represents the most common acid–base abnormality encountered pre‐dialysis and has several adverse effects [27, 28, 29], respiratory disorders are also frequent [4], and metabolic alkalosis requiring correction by dialysis is also encountered [30, 31]. The serum total carbon dioxide (*TCO*_2_), which is routinely measured in serum along with other electrolytes, constitutes an inadequate indicator of the acid–base status pre‐hemodialysis [32, 33, 34, 35]. Metabolic alkalosis is encountered in uremic patients and can be treated by dialytic methods [33, 36]. Blood gas analysis should be performed [33, 36].

A hypothetical case requiring correction of both severe hyponatremia and metabolic acidosis using the four stream system of this report is presented. A patient having missed several sessions of hemodialysis presents with seizures, coma, serum sodium of 100 mmol/L, arterial blood pH of 7.21, arterial bicarbonate of 8 mmol/L and arterial *P*_*CO*2_ of 20 mmHg. A slow hemodialysis session with this new method is planned with targets of 108 mmol/L for serum sodium and 25 mmol/L for arterial bicarbonate. Serum glucose, protein, and lipid concentrations are normal allowing an estimate of serum water content at 93% and of sodium concentration in serum water of 100/0.93 = 107.5 mmol/L. An interstitial fluid to‐plasma water Gibbs–Donnan coefficient of 0.950 has been used for cation concentrations [37]. Using the same coefficient for dialysis fluid‐to‐plasma water sodium concentrations, the dialysis fluid sodium concentration value required for a serum sodium of 108 mmol/L (sodium concentration in serum water of 108/0.93 = 116.1 mmol/L) is 0.950 × 116.1 = 110.3 mmol/L. The ***W***: ***S***: ***B***: ***A*** flow rate ratio of 41.80:1.04:1.16:1 is obtained by entering 25 for dialysis fluid bicarbonate concentration and 110.3 for dialysis fluid sodium concentration in the EXCEL flow sheet. Thus each 45 L of dialysis fluid consists of 41.80 L of product water, 1.04 L of concentrate ***S***, 1.16 L of concentrate ***B***, and 1 L of concentrate ***A***.

Measurements in the dialysis fluid reveal a pH of 6.86, a dialysis fluid sodium of 110.5 mmol/L, and a dialysis fluid bicarbonate of 25 mmol/L. The relation between the sodium concentrations in serum and serum water used applies only when serum water content is 93%, the Gibbs–Donnan coefficient used represents an average estimate, and the change in serum bicarbonate concentration cannot always be predicted with accuracy. These reasons dictate the potential need of changing the dialysis fluid composition by monitoring the concentrations of sodium and bicarbonate (or *TCO*_2_) in serum during the hemodialysis session.

## 4 Discussion

It is important to emphasize that this is a proof‐of‐concept report. Confirming the feasibility and applicability of the method presented in clinical practice requires in vitro or ex vivo testing. The method presented in this report addresses the correction by hemodialysis of cases of dysnatremias and/or metabolic acid–base disorders using specific desired concentrations of sodium and bicarbonate in the dialysis fluid. Single‐patient dialysis fluid delivery systems are envisioned. This new method is designed to allow the clinical application of the dialysis system proposed. As noted in the Introduction, the methods presented in the two previous publications on the use of the new dialysis system [11, 12] do not allow the calculation of specific desired values of sodium and bicarbonate in the dialysis fluid.

Monitoring of the serum sodium concentration during hemodialysis sessions that treat dysnatremia cases should guide changes in the dialysis fluid sodium concentration. The accuracy of sodium concentration in the dialysis fluid should also be addressed. In one study, only 57% of the measured dialysis fluid sodium values were within 2 mmol/L of the prescribed values [38]. Sodium concentration in the dialysis fluid should be measured after each change of the composition of dialysis fluid. The rate of decrease in blood urea concentration merits consideration in addition to the sodium in the dialysis fluid. Hemodialysis causes brain cells to swell during rapid decreases in blood urea concentration [39, 40]. Decreasing the blood urea concentration slowly combined with the rise in serum sodium concentration will prevent osmotic swelling of the brain cells. The hemodialysis prescription should combine a proper dialysis fluid sodium concentration and slow dialysis by lowering blood and/or dialysis fluid flow rates, using a smaller dialyzer, shortening of the dialysis session time, or a combination of these measures [23]. Repeated hemodialysis sessions on the same day may be needed to bring the serum sodium concentration to its desired level and to remove azotemic compounds adequately.

The optimal rate of correction of serum bicarbonate by hemodialysis in metabolic acidosis remains challenging. Slow correction may be preferable. Correction of metabolic acidosis leads to a rise in body fluid *P*_*CO*2_. During rapid correction, the transfer rate of carbon dioxide between blood and cerebrospinal spinal fluid (CSF) exceeds that of bicarbonate, resulting in CSF *P*_*CO*2_ levels close to those in arterial blood, and low CSF bicarbonate and pH levels leading to the development of neurological manifestations [41]. In dogs with acute uremia, rapid hemodialysis caused decrease of pH in both the cerebral cortex and cerebrospinal fluid, and cerebral edema, whereas these changes did not develop in dogs treated by slow hemodialysis [42]. The severity and type of metabolic acidosis should also be considered when choosing the target bicarbonate concentration in the dialysis fluid. The amount of sodium bicarbonate required to result in an equivalent increase in serum bicarbonate concentration is higher in severe than in moderate metabolic acidosis [43]. Overproduction metabolic acidosis (e.g., lactic acidosis) requires special attention, because the acid–base status of the patient is determined by removal, continuous acid production, and bicarbonate supplementation during hemodialysis. Slow and short dialysis, evaluating the acid–base status of the patient during the dialysis session, and changing the concentration of bicarbonate in the dialysis fluid during the session if needed are required.

Repeated hemodialysis sessions over 24 h provide the most appropriate way to correct combined severe dysnatremias and metabolic acid–base disorders. The procedure should be performed in an Intensive Care Unit under continuous clinical observation by experienced medical and nursing personnel. Hypervolemic or hypovolemic dysnatremia require additional measures. Ultrafiltration for hypervolemia tends to increase the serum sodium concentration [44]. Hypovolemic dysnatremia requires saline infusion. The saline infused should have a sodium concentration equal to the dialysis fluid sodium concentration. This is achieved by adding 5% dextrose solution to isotonic saline when treating hyponatremia. For example, if dialysis fluid sodium equals 110 mmol/L, the infusate sodium concentration of 110 mmol/L is obtained by combining 0.705 L of isotonic saline, which contains 154 mmol/L of sodium, and 0.295 L of 5% dextrose. During hemodialysis sessions treating dysnatremias combined with metabolic acid–base disorders, the concentrations of sodium and bicarbonate in the dialysis fluid, the pH of the dialysis fluid, and serum electrolytes and acid–base parameters should be measured after each modification of the dialysis fluid. The serum measurements should be repeated after the end of the procedure. Point‐of‐care application of these measurements plays an important role. Correction of dysnatremias by continuous renal replacement treatment (CRRT) methods requires estimates of body water for the composition of the dialysis fluid [44]. One advantage of the four‐stream hemodialysis system is that it does not require an estimate of total body water for this function.

Another potential application of the proposed system is in clinical trials for identifying optimal standards for sodium and bicarbonate concentrations in the dialysis fluid. Further investigations for the optimal values of dialysis fluid sodium [45, 46, 47, 48] and bicarbonate [49, 50, 51, 52] are required. Individual control of the components of the hemodialysis [53] and hemodiafiltration [54] fluid, including sodium and bicarbonate, was also proposed. The system presented in this report can facilitate individualization of dialysis fluid sodium and bicarbonate concentrations.

In conclusion, the novel method presented in this report has the potential of treating severe dysnatremias and metabolic acid–base disturbances. Absence of clinical experience represents the main limitation of this report.

## Conflicts of Interest

The authors declare no conflicts of interest.

## Supporting information


**Data S1** EXCEL flow sheet providing the ***W***: ***S***: ***B***: ***A*** flow rate ratio for any desired combination of dialysis fluid concentrations of bicarbonate ([*HCO*_3_]_df_) and sodium ([*Na*]_df_).
Use of this EXCEL flow sheet consists of writing in the proper columns the desired [*HCO*_3_]_df_ and [*Na*]_df_ values and clicking on the sign “Press Ctrl m to Modify Columns A to C.” The EXCEL flow sheet will then report the desired flow rates of streams ***W***, ***S***, and ***B*** and the concentration of chloride in the final dialysis fluid resulting from these flow rates and a flow rate of stream ***A*** equal to 1.0. The sodium concentration in the dialysis fluid provided by stream ***A*** is 63 mmol/L in this EXCEL flow sheet. The value reported in column “***S*** flow” will be negative when the combination of desired [*HCO*_3_]_df_ and [*Na*]_df_ is outside the limits of the ranges allowed by this system.

## Data Availability

Data sharing is not applicable to this article as no new data were created or analyzed in this study.
